# Failure Handling of Robotic Pick and Place Tasks With Multimodal Cues Under Partial Object Occlusion

**DOI:** 10.3389/fnbot.2021.570507

**Published:** 2021-03-08

**Authors:** Fan Zhu, Liangliang Wang, Yilin Wen, Lei Yang, Jia Pan, Zheng Wang, Wenping Wang

**Affiliations:** ^1^Department of Computer Science, The University of Hong Kong, Hong Kong, Hong Kong; ^2^Department of Mechanical Engineering, The University of Hong Kong, Hong Kong, Hong Kong; ^3^Department of Mechanical and Energy Engineering, Southern University of Science and Technology, Shenzhen, China

**Keywords:** soft robot applications, pick and place, failure handling, visual tracking, proprioception

## Abstract

The success of a robotic pick and place task depends on the success of the entire procedure: from the grasp planning phase, to the grasp establishment phase, then the lifting and moving phase, and finally the releasing and placing phase. Being able to detect and recover from grasping failures throughout the entire process is therefore a critical requirement for both the robotic manipulator and the gripper, especially when considering the almost inevitable object occlusion by the gripper itself during the robotic pick and place task. With the rapid rising of soft grippers, which rely heavily on their under-actuated body and compliant, open-loop control, less information is available from the gripper for effective overall system control. Tackling on the effectiveness of robotic grasping, this work proposes a hybrid policy by combining visual cues and proprioception of our gripper for the effective failure detection and recovery in grasping, especially using a proprioceptive self-developed soft robotic gripper that is capable of contact sensing. We solved failure handling of robotic pick and place tasks and proposed (1) more accurate pose estimation of a known object by considering the edge-based cost besides the image-based cost; (2) robust object tracking techniques that work even when the object is partially occluded in the system and achieve mean overlap precision up to 80%; (3) contact and contact loss detection between the object and the gripper by analyzing internal pressure signals of our gripper; (4) robust failure handling with the combination of visual cues under partial occlusion and proprioceptive cues from our soft gripper to effectively detect and recover from different accidental grasping failures. The proposed system was experimentally validated with the proprioceptive soft robotic gripper mounted on a collaborative robotic manipulator, and a consumer-grade RGB camera, showing that combining visual cues and proprioception from our soft actuator robotic gripper was effective in improving the detection and recovery from the major grasping failures in different stages for the compliant and robust grasping.

## 1. Introduction

**Figure 1:**
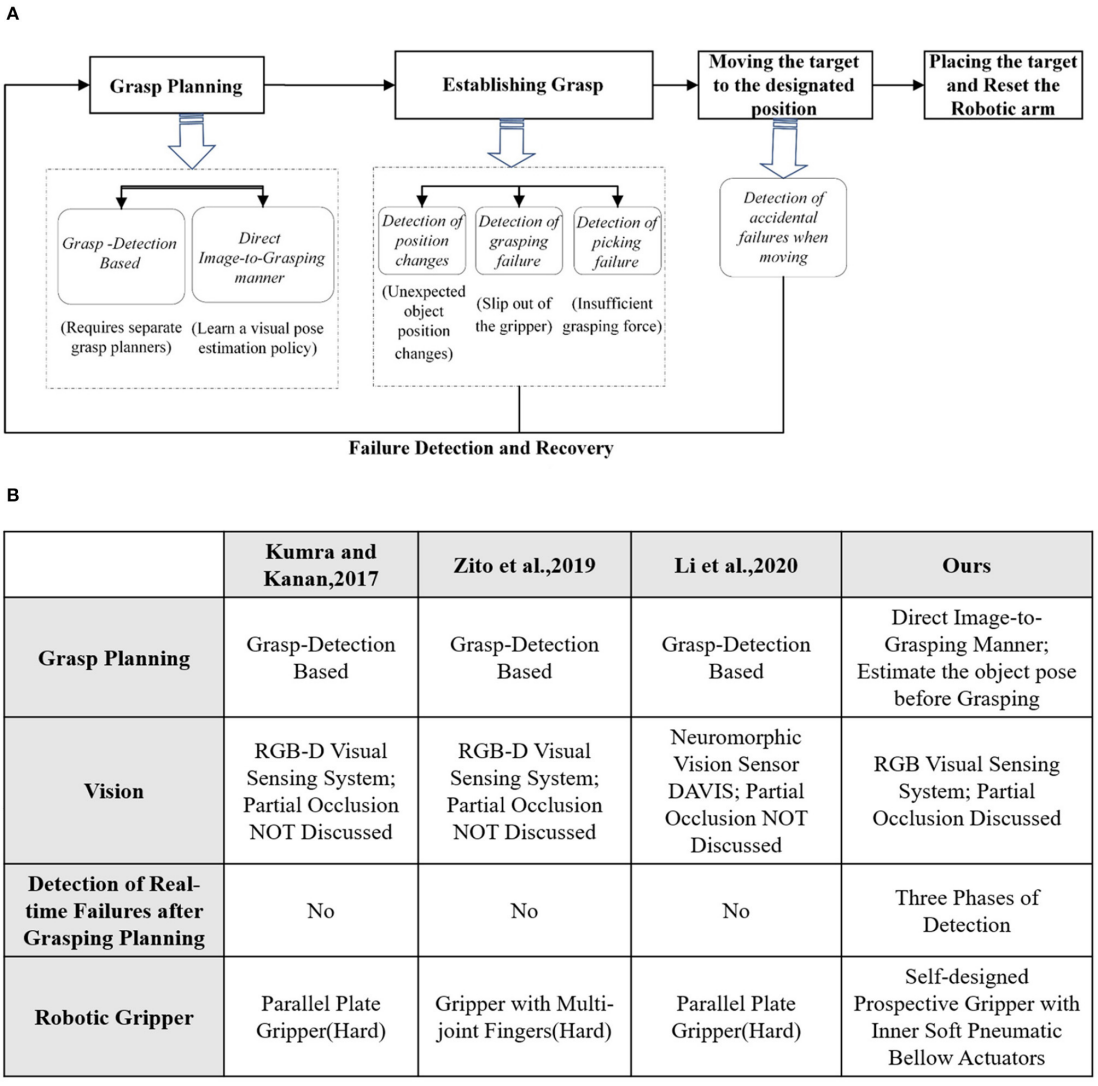
**(A)** The entire procedure of robotic pick and place task. **(B)** Comparative analysis with other methods.

The success of a robotic pick and place task depends on the success of the entire procedure: from the planning phase (object detection and grasp planning), to the grasping phase (actually establishing the grasp), to the lifting and moving phase (transit the object toward target site), and the final releasing phase (descending the object and release the grasp). Being able to detect and recover from grasping failures throughout the entire process is therefore a critical requirement for both the robotic manipulator and the gripper (see Figure 1).

Grasp planning aims at generating better grasping proposals to improve the success rate of robotic grasping. It can be categorized as grasp detection based (Kumra and Kanan, 2017; Zito et al., 2019; Li et al., 2020) and direct image-to-grasping manner. The former mainly generates the grasping proposals for the novel objects and it utilizes grasping contacts to compensate for the pose uncertainty. The latter detects structured grasp representations from images by the pose estimation of a known object (Sundermeyer et al., 2018).

When establishing grasp and moving the target to the destination, it is critically important to detect and recover from any accidental failure in real scenarios. Even with an excellent grasp planning, unexpected failure may still occur in the pick and place task due to environmental changes or intrinsic systematic errors. Without an effective failure detection and recovery mechanism, the robotic system may crack accidentally and be less efficient.

Visual servoing (Cowan et al., 2002; Kragic et al., 2002) was popular for guiding the above phases in the robotic system. Some typical object tracking algorithms (Grabner et al., 2006; Bolme et al., 2010; Kalal et al., 2010) have been well studied. Li et al. (2020) built a sensing pipeline through a neuromorphic vision sensor DAVIS to satisfy the real-time features in object detection and tracking. However, preserving visibility of the target has been the key to robust object tracking in these algorithms and the performance of algorithms becomes much weakened when the partial occlusion exists. Robust tracking techniques under the partial object occlusion are of great significance to a robust robotic grasping system.

Meanwhile, under-actuated robotic grippers (Zhou et al., 2017) recently tend to have a variety of advantages over the rigid-bodied counterparts when the gripper is interacting with the environment. There are numerous grippers with novel designs of compliant mechanisms, working as both actuators and sensors to generate movement and provide proprioceptive feedback simultaneously (Su et al., 2020; Zhou et al., 2020). Endowing soft robotic grippers with proprioception enables reliable interactions with environment.

In this paper, we aim to investigate an effective grasping system from the beginning to the endpoint, by considering the partial object occlusion as a normal condition. We especially focus on the failure detection and recovery framework in the grasping system by combining the specific proprioceptive capability of our soft gripper and the visual cues from the highly obstructed view when the failure occurs. The proprioceptive soft gripper used in the paper was developed in our recent work (Wang and Wang, 2020). It was pneumatically driven by soft bellows actuator and the pressure of the actuator was leveraged for sensing the gripper movement and external contact (see **Figure 5**). The main contributions and novelties are listed as follows:

(1) more accurate pose estimation of a known object by considering the edge-based cost besides the image-based cost;
(2) robust object tracking techniques that work even when the object is partially occluded in the system and achieve mean overlap precision (OP) up to 80%;
(3) contact and contact loss detection between the object and the gripper by analyzing internal pressure signals of our gripper;
(4) robust failure handling of robotic pick and place tasks with the combination of visual cues under partial occlusion and proprioceptive cues from our soft gripper to effectively detect and recover from different accidental grasping failures.

## 2. System Architecture

### 2.1. System Modeling

**Figure 2:**
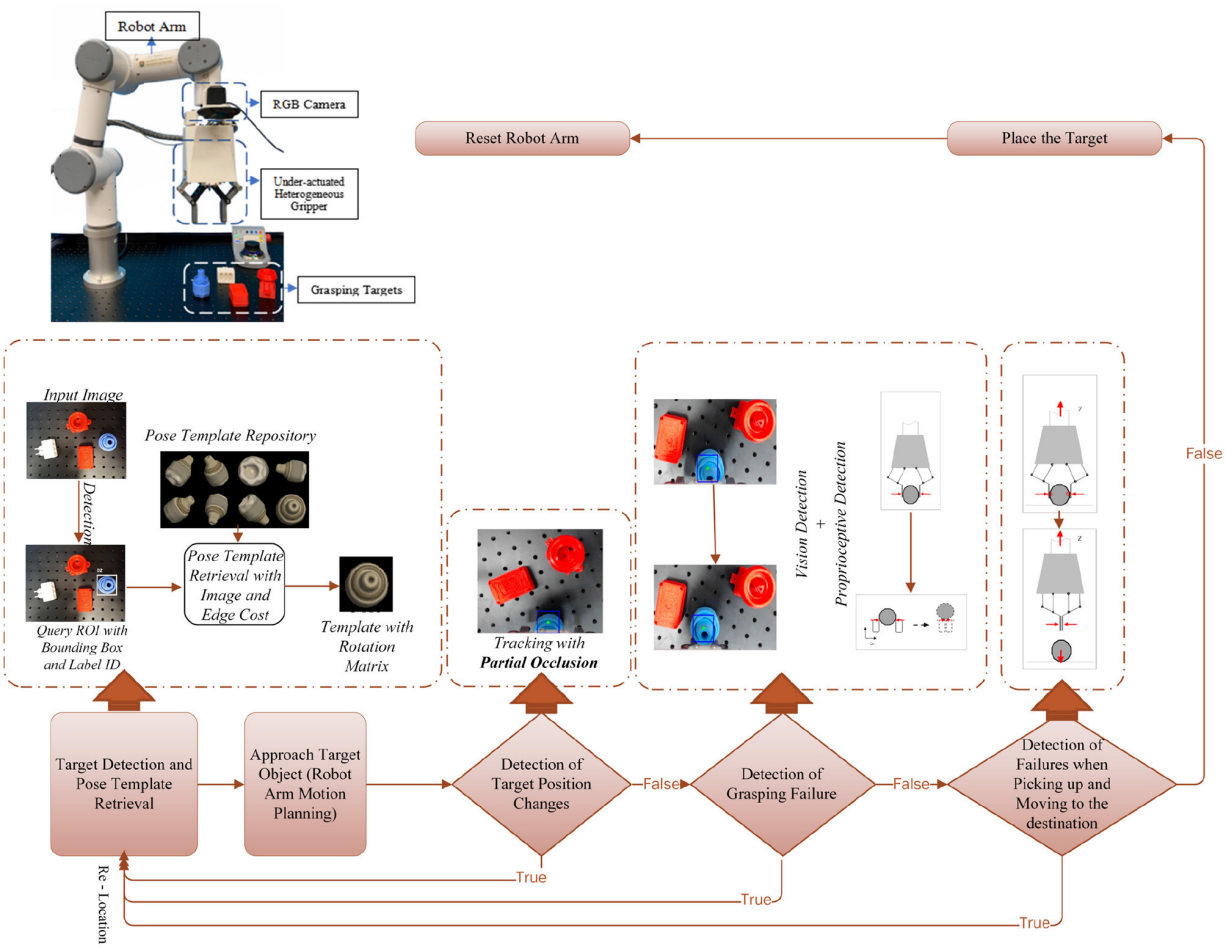
The top-left is the setup of the proposed robotic pick and place platform, with a collaborative robotic arm and a proprioceptive soft robotic gripper with inner soft pneumatic bellow actuators backed by rigid frames, and pressure sensors for monitoring bellow's inner pressure. A consumer-grade RGB camera is used for visual detection and tracking. The bottom is the proposed system pipeline with feedbacks of both visual and proprioceptive cues.

The setup we considered consists of an RGB camera and a proprioceptive gripper, which are equipped on the robot arm. The robot arm is controlled by an operator acting on a master device and interacting with the environment by combining the proprioception of our soft gripper (Wang and Wang, 2020) and the visual cues from the camera view. The relative coordinates of the camera and the testbed are first calibrated, and the depth is accordingly computed. We assume all the objects are put on the same testbed. The system setup is illustrated in Figure 2.

Our system first performs automatic target detection and poses estimation based on an RGB image and an edge map. Then a robust object tracking algorithm continuously works to provide real-time visual cues for failure detection and recovery, even if the object is highly obstructed in the camera view. Meanwhile, the proprioceptive capability of our soft gripper (Wang and Wang, 2020) is utilized in the system to sense the contact between the object and the gripper. We measure the actuation pressure in the soft actuator chambers to extract the external contact force and further reflect the contact status between the gripper and object. The proprioceptive capability is combined with visual cues to guarantee the effectiveness of our system.

### 2.2. Workflow Illustration

The proposed multi-sensor collaboration architecture aims at facilitating effectiveness of failure detection and recovery in the grasping. A general illustration of the system pipeline is shown in Figure 2.

The input of our system is the starting frame recorded by the in-hand camera. We first aim at target detection and pose estimation. The target is first assigned by the user and denoted as the number corresponding to the predefined template. Then the target is detected on the query image and the target's pose is estimated by template retrieval with an image and edge cost. For the determination of the target's pose, previous work (Zhou et al., 2017) prefer to first detect objects without recognition. Then a planner, such as MoveIt planner (Coleman et al., 2014), is implemented to generate multiple motion plans and intuitively determine the pose. Compared with our previous work (Zhou et al., 2017), pose estimation in this paper is more efficient based on object recognition. Because any accidental changes or failures affect subsequent steps in grasping, we design three phases of detection by combining the visual and proprioceptive cues to improve the effectiveness of our system. The first detection is designed for disturbance from external factors. For example, the target may be accidentally moved as the gripper is approaching. Our system detects position changes with the proposed object tracking algorithm. It can still robustly work in the challenging scenario that the target is partially occluded by the gripper in the camera view. If the position change of target is not detected in visual tracking, a grasping trial will be executed. Otherwise, if the target is moved, our system will relocate and track the target in the current camera view. If the target is reported lost in the current view, the arm will be reset. Target detection and pose estimation will be executed in the new camera view. The second detection aims at checking if the last grasping trial is successful. The failure here usually results from internal disturbance, such as the inaccurate pose of the gripper in the former trial. Both visual and proprioceptive cues are utilized by observing whether the coordinate of the target remains the same and the force changes measured by inner pressure sensor have followed the common rules during the grasping trial. Then combined feedbacks will guide the determination of the system. If no failure happens, the system will step into the next phase. Otherwise, the system will timely go back to the very beginning phase. Compared with our previous work (Zhou et al., 2017) without timely failure detection in grasping, the combination of visual and proprioceptive information contributes to the effectiveness of failure reaction in our system. The third detection aims at checking picking failure based on the proprioceptive information. Proprioceptive cues can be sensitively observed from the embedded air-pressure sensor of our soft gripper. Thus, the soft gripper has its specific advantages in our case besides its compliance advantage in grasping. Through simple data processing, the contact force between the object and gripper can be estimated. Picking failure occurs when a sudden decrease in the estimated contact force is detected. In the final phase, if object picking succeeds, the target will be placed in the expected position and the robot arm will be reset.

## 3. Methodology

This section clarifies some technical details in our system. It can be divided into three parts. In the first part, we introduce how to automatically detect the target and estimate the pose of the target by the template retrieval. Besides the canonical image-based cost, we introduce the edge-based cost to improve the accuracy of object pose estimation. In the second part, we present the target tracking algorithm that can robustly work even with partial object occlusion. In the third part, we explore the details about the proprioceptive of our soft gripper in the failure detection and recovery system.

### 3.1. Object Detection and Pose Template Retrieval

**Figure 3:**
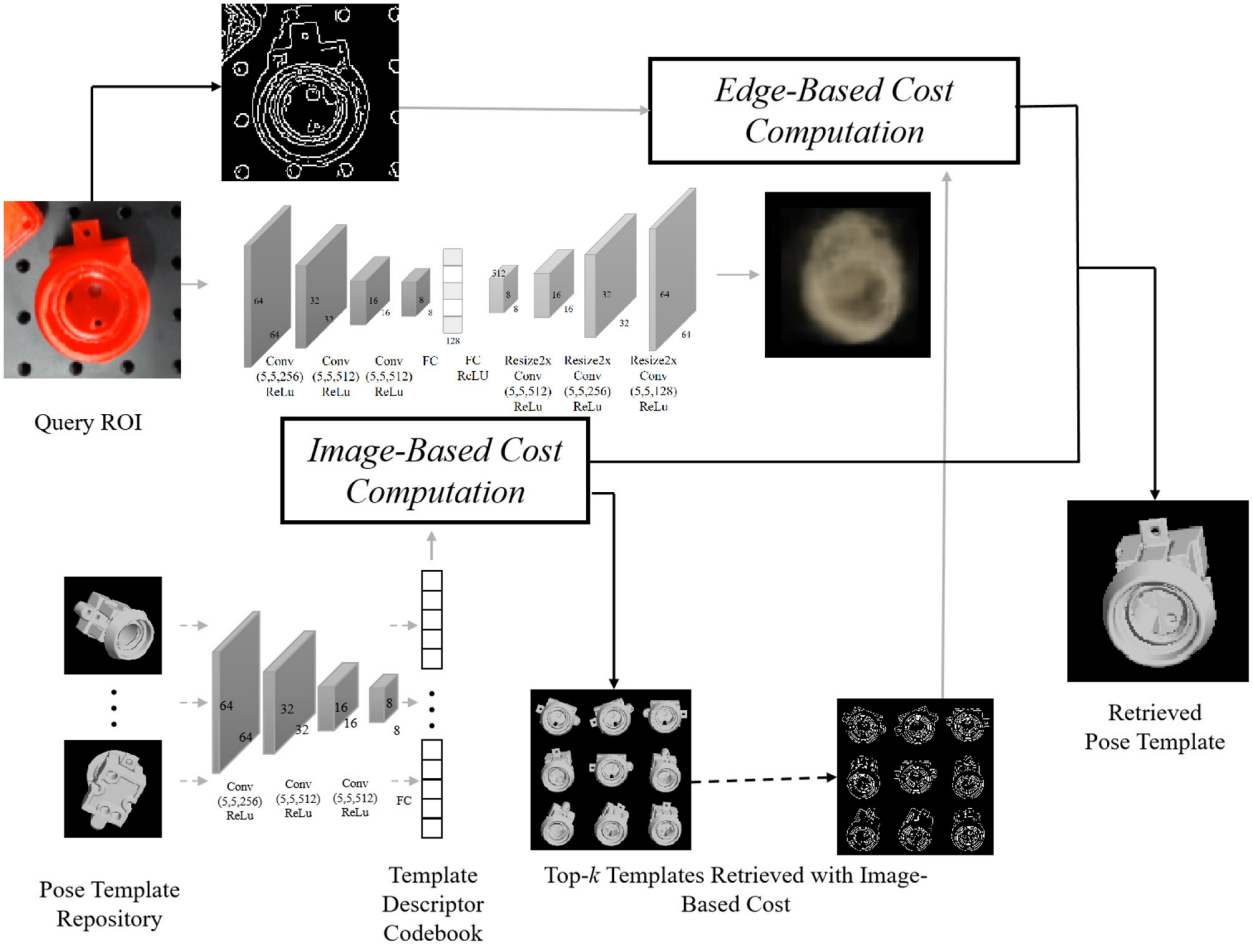
The pipeline for pose template retrieval, where we first compute the image-based descriptor based on the reconstruction of the foreground model and use the image-based cost to select top-*k* template candidates. Among these *k* candidates, the edge-based cost is then computed with the corresponding edge map (see details in section 4.1), followed by a combination of two scores to re-rank. Off-line computation for templates is annotated by dash lines, and online computation by solid lines.

In this part, we illustrate our algorithm for object detection and pose template retrieval. As illustrated in Figure 3, we implement the network described in Sundermeyer et al. (2018) to compute the image-based cost by first finetuning Single Shot MultiBox Detector (Liu et al., 2016) on synthetic images to help detect and label objects on the query image, and then reducing the pose estimation issue to pose template retrieval, in which we create a pose repository for each object by rendering clean images with different views and inner plane rotations. To retrieve the best pose template from this repository, here we innovatively combine not only the state-of-art work (Sundermeyer et al., 2018) with a deep neural network, but also a canonical edge-based cost (Shotton et al., 2008) to improve robustness. Figure 3 shows how we combine these two cues for the pose template retrieval problem, while in the following paragraphs we will illustrate these two costs and the way we combine them for the pose template retrieval in detail.

#### 3.1.1. Image-Based Cost

Through the supervised process of reconstructing the object's appearance in the RGB image while eliminating the influence of background clutter, occlusion, geometric, and color augmentation, Sundermeyer et al. (2018) output a descriptor that conveys the 3D orientation information. By looking up the descriptor codebook for poses in the repository, a cosine similarity cost is computed to measure the similarity between the query Region Of Interest (ROI) and the *i*th pose template in the repository:

(1) $$\begin{array}{l} C_{i}^{IMG}=-\frac{z_{q}^{T}z_{i}}{\left\|z_{q}^{T}\|\cdot \|z_{i}\right\|} \end{array}$$

where *z*_*q*_,*z*_*i*_ ∈ *R*^128^ correspond to the computed descriptors for the query ROI and *i*th pose template in the repository, respectively. Here, we take a negative to ensure a smaller cost indicates a better matching.

#### 3.1.2. Edge-Based Cost

We utilize oriented Chamfer distance (Shotton et al., 2008) to compute the edge-based cost. With a given edge map and the set of edge points *T*_*i*_ for the *i*th pose template, we define the nearest query edge point V(t) for *t*∈*T*_*i*_ as:

(2) $$\begin{array}{l} V\left(t\right)=\underset{q\in Q}{\mathrm{argmin}}\|q-t{\|}_{1} \end{array}$$

where *Q* indicates the set of edge points from the query ROI, and *L*1 distance is used. So we evaluate the edge-based cost:

(3) $$\begin{array}{l} C_{i}^{EDGE}=\frac{1}{\left|T_{i}\right|}\underset{t\in T_{i}}{\sum }\|V\left(t\right)-t{\|}_{1}+\lambda \|\phi \left(V\left(t\right)\right)-\phi \left(t\right)\| \end{array}$$

where |*T*_*i*_| indicates the cardinality of the set *T*_*i*_ and ϕ(*x*) is the orientation of edge at edge point *x*. *lambda* is the weighting factor that balances the distance and orientation differences.

#### 3.1.3. Enhanced Image-and-Edge Costs

Due to the gaps between synthetic training data and real test images in terms of environments and model precision, the image-based cost may fail to retrieve the correct pose reasonably, while the edge-based cost is robust under these changes. Thus, we first use image-based costs to provide top-*k* pose candidates. Then we use a weight parameter μ(0 ≤ μ ≤ 1) to linearly combine both image and edge-based costs to re-rank these *k* candidates:

(4) $$\begin{array}{l} C_{i}=\mu C_{i}^{IMG}+\left(1-\mu \right)C_{i}^{EDGE} \end{array}$$

### 3.2. Visual Tracking Under Partial-Occlusion Circumstance

In this section, we address the case of continuously tracking the target even though partial occlusion occurs. We use correlation filters to model the appearance of the target and perform robust tracking via convolution. Recently, correlation-filters-based trackers (CFTs), which were widely used in recognition (Savvides et al., 2004) and detection (Bolme et al., 2009), have shown promising performance in object tracking. The CFTs estimate the target's position by correlation filters with different kinds of features. In the Fourier domain, the correlation score is computed by the element-wise multiplication between image features and the complex conjugate of the correlation filter (Bolme et al., 2010). Inverse fast Fourier transform (IFFT) is utilized to transform the correlation back to the spatial domain. The peak correlation score indicates the target's center.

**Figure 4:**
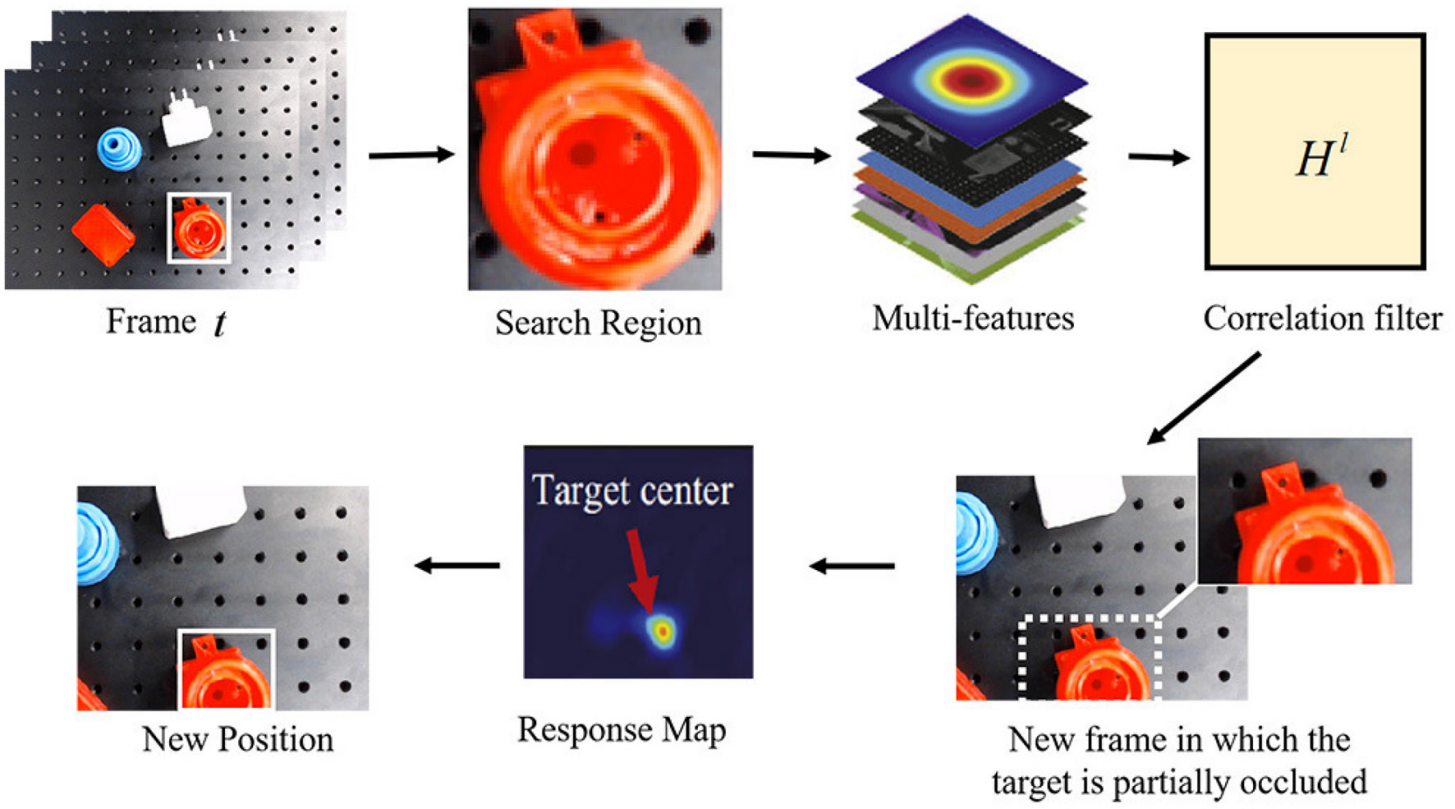
Illustration of the proposed robust correlation-filters-based tracking method for **partial occlusion**.

A general illustration of the tracking method, which is feasible when partial occlusion exists, is shown in Figure 4. Let *f* denotes the feature of an image patch and *g* denotes the desired output, we can get the correlation filter in the Fourier domain (Bolme et al., 2010). The state of the target can be estimated by learning a discriminative correlation filter (DCF) *h*, which is trained by an image patch *I* of size *M* × *N* around the target. The tracker considers all circular shifts $f_{m.n}^{l}$,(*m, n*) ∈ 0, ..., *M* − 1 × 0, ..., *N* − 1 as features of training patches for training correlation filters, where *l* ∈ 1, ..., *d* is the dimension of features. The correlation filter *h*^*l*^ of each feature is built by minimizing a cost function as follows:

(5) $$\begin{array}{l} h^{*}=\underset{h}{\mathrm{argmin}}\underset{m,n}{\sum }\|\overset{d}{\underset{l=1}{\sum }}f_{m,n}^{l}⊙h^{l}-g\left(m,n\right){\|}^{2} \end{array}$$

where ⊙ symbol denotes circular correlation. All the training patches are selected from I by dense sampling. Equation (5) is a linear least square system that transforms tasks from the spatial domain into the frequency domain with a simple element-wise relationship. The Fourier transform of the input image, the filter, and the output can be represented by *F*^*l*^, *H*^*l*^, and $G_{i},\bar{F^{l}},\bar{F^{l}}$ represent the complex conjugation operations, and above minimization problem takes the form:

(6) $$\begin{array}{l} \underset{H^{*}}{\min}\underset{i}{\sum }|{\bar{F}}^{l}H^{l}-G_{i}{|}^{2} \end{array}$$

By solving for *H*^*l*^, a closed-form expression is shown as:

(7) $$\begin{array}{l} H^{l}=\frac{\sum_{i}\bar{G_{i}}F^{l}}{\sum_{i}\bar{F^{l}}F^{l}} \end{array}$$

To estimate the target's position in the frame *t*, a new patch *z* with size *M* × *N* will be cropped out according to the target's position in the frame *t* − 1. Based on the correlation filter, the response output is then computed and transformed back into the spatial domain by IFFT. The location of the maximum value in the response output indicates the shifted center of the target from frame *t* − 1 to frame *t*.

#### 3.2.1. Tracking With Partial Occlusion

The algorithm performs well under scale variation and partial occlusion. The Peak-to-Sidelobe ratio, which measures the strength of a correlation peak, splits the response of the filter into the maximum value and the “side lobe” that consists of the rest of pixels in the region, including a small window (i.e., 11 × 11) around the peak. If the occlusion is detected, the tracker should attempt to hallucinate the target until it can be detected again. For occlusion solving, we divide the target into several patches and then compute the Peak-to-Sidelobe ratio of every response map. According to the maximum in the response map, the partially occluded target can be tracked robustly. The occlusion detection and solving techniques ensure the tracker to work robustly and reliably in robotic grasping.

### 3.3. Proprioceptive Grasp Failure Detection

**Figure 5:**
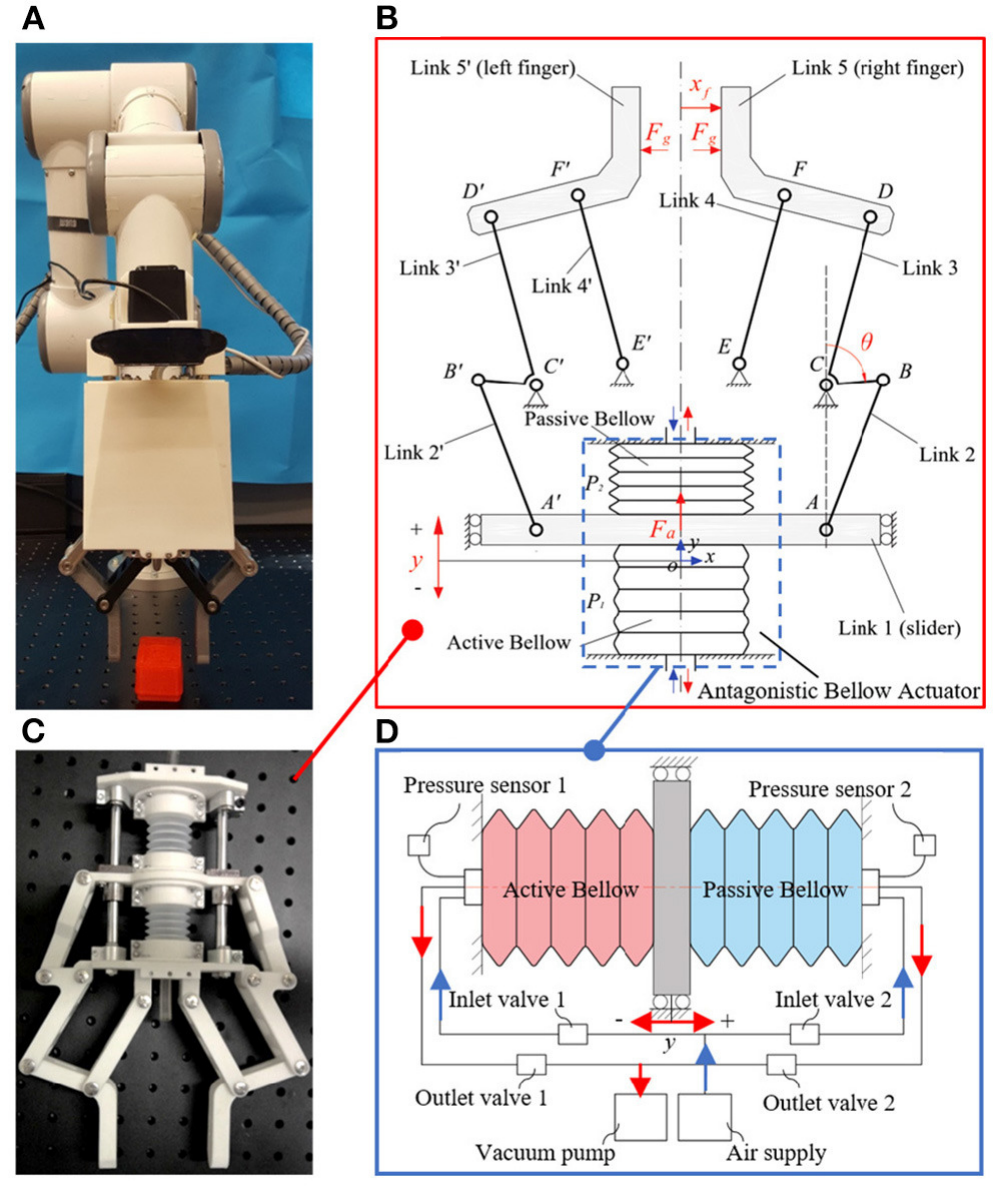
Proprioceptive gripper with proprioceptive sensing capability: **(A)** prototypical setup of the proprioceptive gripper; **(B)** mechanism of the proprioceptive gripper; **(C)** internal gripper structure showing the bellows soft actuator; **(D)** schematic of the pneumatic bellow actuator.

Object picking and placing tasks are a series of contact involving forces, which cannot be easily monitored by vision. Vision can indicate to the robotic system the position of the target object, but it requires physical contact feedback to fast detect-response to dynamical changes and enable robust grasping. In this section, based on the soft actuated rigid gripper developed in the previous work (Wang and Wang, 2020), we proposed a contact and contact loss monitoring method for the grasp failure detection in the pick-and-place task. Figure 5 presents the prototype and mechanism of the soft actuated rigid gripper. The soft actuated rigid gripper was constructed with antagonistic bellows and plane six-bar linkages and is pneumatically operated with a simple control system. Two pressure sensors were used for monitoring bellows' inner pressure. We did not attach any traditional force or position sensors on the soft actuated rigid gripper but to leverage the pressure signal of the soft bellows actuators for estimate the joint movements and external contacts. In such configuration, this soft actuated rigid gripper is endowed with so-called proprioceptive capability.

#### 3.3.1. Contact Force Estimation

The contact force at the fingertip was proposed to be estimated by a generalized momentum observer (Wang and Wang, 2020). The observer dynamics is given by

(8) $$\begin{array}{l} r=K_{o}\left(M\left(\theta \right)\overset{∙}{\theta }-\int_{0}^{t}\left(\left(F_{a}-k_{a}y\right)\frac{\partial y}{\partial \theta }+C\left(\theta ,\overset{∙}{\theta }\right)-g\left(\theta \right)+r\right)ds\right) \end{array}$$

where the monitoring signal *r* is observer output,*K*_*o*_ is observer gain. Displacement of the actuator y and link angle θ is a set of generalized coordinates to formulate the dynamic model of the gripper system. *k*_*a*_ is the axial stiffness of the actuator, which was theoretically and experimentally calibrated as a constant. The actuation force *F*_*a*_ is estimated by the measured pressures *P*_1_ and *P*_2_ of the active and passive bellow, that is *F*_*a*_ = *A* · (*P*_1_ − *P*_2_), where *A* is the effective active area of the air. *M*(θ) is the mass inertia, $C\left(\theta ,\overset{∙}{\theta }\right)$ is the centrifugal and Coriolis force, and g(θ) is the gravitational torques in the link joints. Detailed deduction of the momentum observer can be seen in Wang and Wang (2020). The contact force at the fingertip *F*_*g*_ can then be estimated via the observer output as

(9) $$\begin{array}{l} F_{g}={\left(\frac{\partial x_{f}}{\partial \theta }\right)}^{-1}r \end{array}$$

where *x*_*f*_ is the displacement of the gripper finger.

#### 3.3.2. Contact Detection

To detect the physical contact between gripper fingertips and the object, a contact detection function *cd*(·) can be introduced to map the estimated contact force *F*_*g*_(*t*) into the two classes *TRUE* or *FALSE*:

$$cd:F_{g}(t)\to \{TRUE,FALSE\}$$

Ideally, the binary classification is obtained by

(10) $$\begin{array}{l} cd\left(F_{g}\left(t\right)\right)=\{\begin{array}{cc} \text{TRUE}, & \text{if}\;F_{g}\left(t\right)\neq 0\\ \text{FALSE}, & \text{if}\;F_{g}\left(t\right)=0 \end{array} \end{array}$$

Considering the error in measurement, modeling, and disturbances, in practice the monitoring signal *F*_*g*_(*t*) ≠ 0 even when no contact occurs. Thus, an appropriate threshold should be considered to obtain a robust contact detection function. Statistical observations of gripper finger open and close motion without grasping any objects, fingertip collision, or external disturbances for a sufficiently long time interval [0, *T*] lead to a definition of μ_*max*_ = *max*{|μ(*t*)|, *t* ∈ [0, *t*]}. Considering a safe margin ε_*safe*_ > 0, the contact detection function can be decided using a conservative threshold σ = μ_*max*_ + ε_*safe*_ :

(11) $$\begin{array}{l} cd\left(F_{g}\left(t\right)\right)=\{\begin{array}{cc} \text{TRUE}, & \text{if}\;F_{g}\left(t\right)\;>\;\sigma \\ \text{FALSE}, & \text{if}\;F_{g}\left(t\right)\leq \sigma \end{array} \end{array}$$

#### 3.3.3. Contact Loss Detection

In case of sudden contact loss due to error in grasping pose configuration, external disturbance, or insufficient contact force, the gripper finger accelerates in the same direction as the grasping force applied to the surface of the object. Therefore, the contact force will suffer a rapid decrease. A binary function can be introduced to recognize contact loss by monitoring the changes in the contact force signal between two suitable time intervals △*F*_*g*_(*kT*) = *F*_*g*_[*NT*] − *F*_*g*_[(*N* − *k*)*T*], where *T* is the sampling time and *kT* is the time interval. Similarly, considering the noise in the estimated contact force, a threshold △ > 0 is used to decide the contact loss detection function *cld*(·)

(12) $$\begin{array}{l} cld\left(△F_{g}\left(kT\right)\right)=\{\begin{array}{cc} \text{TRUE}, & \text{if}\;△F_{g}\left(kT\right)\;<\;△\\ \text{FALSE}, & \text{if}\;△F_{g}\left(kT\right)\geq -△ \end{array} \end{array}$$

### 3.4. Cooperative Work Between Visual Cues and Proprioception of Our Soft Gripper

The object detection and pose estimation algorithm contributes to an initial grasp plan with higher accuracy by considering the edge-based cost besides the image-based cost. Then the tracking algorithm provides the visual cues by robustly reporting the object's real-time position, even if the target is partially occluded in tracking. Systematic failures can be detected from unexpected position changes reflected by the visual cues. Furthermore, immediately after the failure was reported, visual cues can efficiently help the systematic recovery by real-time relocation and pose estimation of the target. Visual cues take effect in both the 1*st* phase (detection of position changes) and 2*nd* phase of detection (detection of grasping failure).

**Figure 6:**
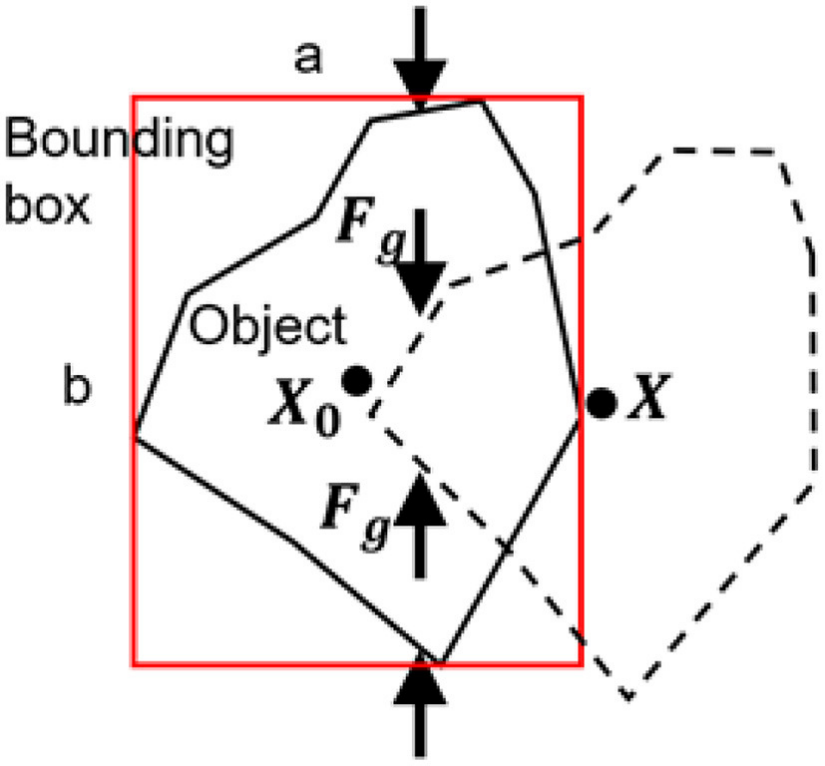
Illustration of grasping failure in the second phase of detection.

Meanwhile, the proprioception of our soft gripper contributes to the contact detection between the gripper and the object by contact force estimation with the internal air pressure sensor. In the 2*nd* phase of detection, see Figure 6, if grasping failure occurs, besides the object position changes reflected by the visual tracking algorithm, the sudden changes of contact force will simultaneously be reported by the internal air pressure. We combine the visual and proprioceptive signal for detection of grasping failure. Let us assume that the maximum contact force during grasping is *F*_*max*_ and after grasping is *F*_*g*_, the object position before grasping is *X*_0_, and the object position after grasping is *X*, and the side length of the rectangle bounding box are *a* and *b*. Intuitively, the object is being stably grasped if larger force *F*_*g*_ retains and *X* is close to *X*_0_ after contact. Using maximum entropy principle, we predict whether contact loss occurs based on the visual or proprioceptive cues and then blend the prediction results for arbitration.

The probability μ_*p*_ of grasping failure predicted by proprioceptive cues can be formulated as

(13) $$\begin{array}{l} \mu_{p}=\frac{e^{-\alpha |F_{g}-F_{max}{|}^{2}}}{e^{-\alpha |F_{g}-F_{max}{|}^{2}}+e^{-\alpha F_{g}^{2}}} \end{array}$$

where α is an adjustable and negative parameter. The probability μ_*v*_ of grasping failure predicted by visual cues can be formulated as

(14) $$\begin{array}{l} \mu_{v}=\{\begin{array}{ll} \frac{e^{-\beta \|X-X_{0}{\|}^{2}}}{e^{-\beta {\left(\sqrt{a^{2}+b^{2}}-\|X-X_{0}\|\right)}^{2}}+e^{-\beta \|X-X_{0}{\|}^{2}}}, & \|X-X_{0}{\|}^{2}<a^{2}+b^{2}\\ 1, & \|X-X_{0}{\|}^{2}\geq a^{2}+b^{2} \end{array} \end{array}$$

where β is an adjustable and negative parameter.

To formulate a confident arbitration, a blending function can be implemented by

(15) $$\begin{array}{l} \mu^{*}=\left(1-\lambda \right)\mu_{p}+\lambda \mu_{v} \end{array}$$

where λ ∈ [0, 1] is a blending factor that represents the confidence on the visual cues or proprioceptive cues for predicting grasping failure. Considering a threshold μ_0_, the grasping failure can be detected by a binary classification.

(16) $$\begin{array}{l} cd\left(F_{g},X\right)=\{\begin{array}{cc} \text{TRUE}, & \text{if}\;\mu^{*}>\mu_{0}\\ \text{FALSE}, & \text{if}\;\mu^{*}\leq \mu_{0} \end{array} \end{array}$$

In the 3*rd* phase of detection, proprioceptive cues are utilized again to inspect the state of picking (see Equation 12). Failures may sometimes occur here because of insufficient grasping force. The detection result in this phase determines whether the internal air pressure needs to be increased.

## 4. Experimental Validation

This section introduces experimental details to validate the outstanding performance of our system. The experimental setup is shown in Figure 2. A consumer-grade RGB camera (Logitech C920) is utilized for object detection and tracking. The proprioceptive robotic gripper provides proprioceptive cues for failure detection. They are both mounted to the end joint of a 6-DoF robot arm (E6, SANTIFICO Ltd.).

### 4.1. Validation of Accuracy Improvement in Object Pose Estimation After Introducing Edge-Based Cost

**Figure 7:**
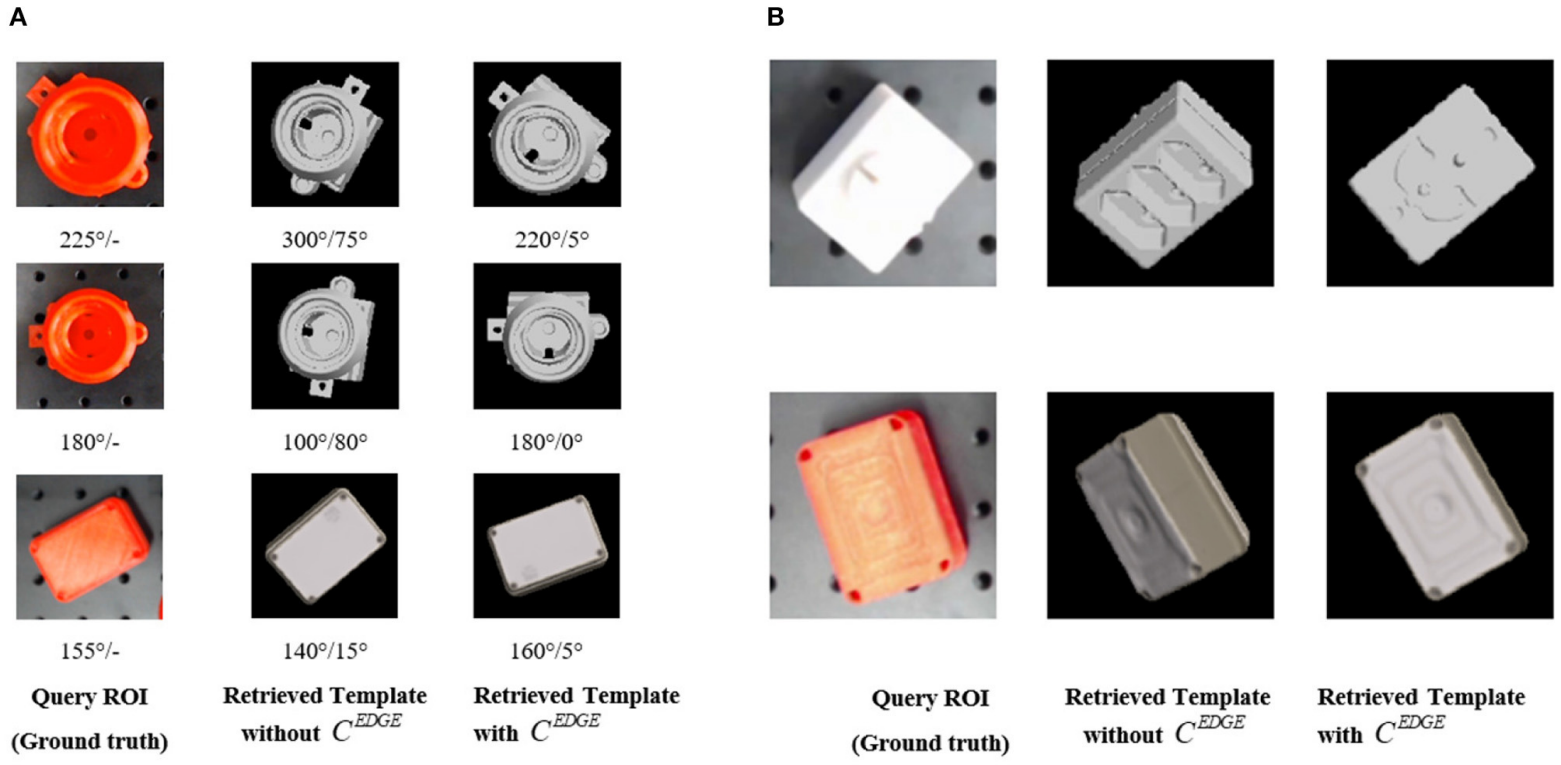
**(A)** Quantitative comparisons that show the improvements brought by the introduction of edge-based cost. The number at the bottom of each row (before “/”) illustrates the rotation angle (in degree) of protruding handles in ground-truth or the estimated pose, as well as (after “/”) the difference between estimated and ground-truth value. The first column is the ground truth of the object pose. The second column is the pose estimated by the method in Sundermeyer et al. (2018), which only considers image-based cost. The third column is the estimated pose of our method considering both image-based cost and the edge-based cost. Our method helps improve the robustness and accuracy of the object pose estimation. **(B)** Qualitative comparisons that illustrate that the introduction of edge cue could help rectify the orientation and provide more accurate pose estimation results. Compared with the color cue, the edge cue is more robust under the variants of lighting conditions.

In experiments of this paper, we use the canonical Canny (1986) to compute edge maps for both pose templates in the repository (off-line computation) and detected query ROIs (online computation). For each object, we generate 3,240 pose templates by evenly sampling the unit sphere space and utilize the image-based cost to select *k* = 20 templates for further re-ranking. We set λ = 10 in edge-based cost and μ = 0.9 for the enhanced image-and-edge cost. To illustrate the advantage of our re-ranking strategy with edge-based cost, we evaluate a model (see Figure 7) with protruding handles, which are crucial for gripping. The image-based cost alone fails to accurately evaluate the orientation of these handles, but the introduction of edge information improves robustness on these detailed but crucial parts.

Figure 7 presents examples with qualitative and quantitative comparisons between two settings that either combines edge information and re-ranking or not. To analyze the estimated result quantitatively, we compute the absolute difference (the error of pose estimation) of rotation angle between estimated and ground-truth pose for the handles referring to the axis perpendicular to the image plane. It is apparently validated that our method, which introduces the edge cost besides the image cost, have advantages over the state-of-art work (Sundermeyer et al., 2018). More reasonable templates are retrieved with the aid of edge-based costs.

### 4.2. Validation of Object Tracking Under Partial Occlusion

Object tracking plays an important role in three phases of detection in our system. However, partial occlusion, which results from the body part of the gripper or external disturbance, may accidentally occur during robotic grasping. As shown in Figure 2, the target is partially occluded by the body part of the gripper. To provide reliable visual guidance for decision-making in real time, we introduce the tracking method that can work robustly even when occlusion exists.

#### 4.2.1. Object Tracking With Partial Occlusion

**Figure 8:**
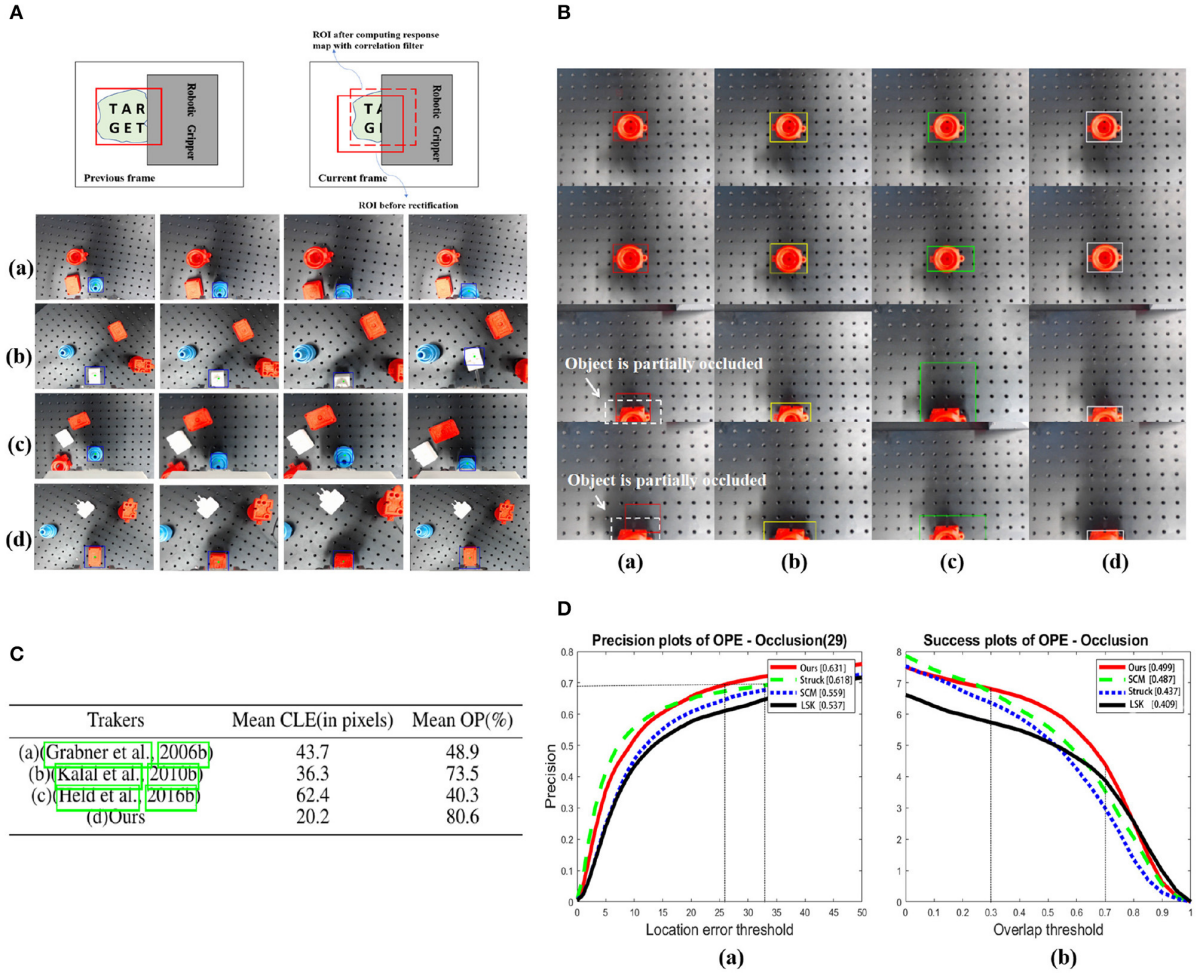
**(A)** Robust object tracking under partial occlusion in different scenarios. *(a)* When no accidental failure occurs. *(b)* Unexpected position changes of the object. *(c)* Grasping failure. *(d)* Picking up failure. Letters “T,” “A,” “R,” “G,” “E,” and “T” are marked on the target to indicate different portions of it. The blue bounding box indicates the position of the target in the camera view. **(B)** Comparison of tracking methods introduced in *(a)* (Grabner et al., 2006), *(b)* (Kalal et al., 2010), *(c)* (Held et al., 2016) with *(d)* ours while the object is partially occluded. The white dashed boxes indicate the object with partial occlusion and bounding boxes in different colors correspond to the results of each tracking method. **(C)** Comparisons between four tracking methods in the same sequence. The mean CLE (in pixels, the lower the better) and OP (%, the higher the better) are presented (when*t*_0_ = 0.5). The best results for the experiments are shown in the bold format. **(D)** Performance evaluation of the proposed method using precision plot and success plot of OPE (One-Pass Evaluation) for sequences having occlusion in OBT-50. In *(a)*, to achieve the same precision at 0.7 or more, our method demands less location error than others. In *(b)*, the partial occlusion(overlap) is usually 30–70% when the gripper approaching to the bottom to grasp the object, and in this overlap interval, our method achieves higher success rate than others.

With numerous systematic tests, the robustness of our tracking algorithm has been obviously reflected, especially when the accidental failures occur. In Figure 8A, we visually presented several tracking examples under different circumstances of our grasping system. Although the object is partially occluded by the body part of our robotic gripper when failures occur, the visual tracking algorithm still robustly provides visual cues to assist the failure recovery of the grasping system.

To demonstrate the advantages of our method over others, we designed the following experiments. With the same experimental setup illustrated in Figure 2, when the target is partially occluded in the camera view, we compare the results of three typical visual tracking algorithms (Grabner et al., 2006; Kalal et al., 2010; Held et al., 2016) in robotic applications with our results. The results are shown in Figure 8B.

To quantitatively evaluate the performance of each tracker, we adopt the evaluation protocol described in Danelljan et al. (2014a,b). (1) Center location error (CLE), which is the average Euclidian distance between the estimated center location of the target and ground truth, and (2) OP, which is the percentage of frames where overlap score is larger than a given threshold *t*_0_ (e.g., *t*_0_ = 0.5). The score is defined as:

(17) $$\begin{array}{l} \text{score}=\frac{\text{area}\left(R_{T}⋂R_{G}\right)}{\text{area}\left(R_{T}⋃R_{G}\right)} \end{array}$$

where *R*_*G*_ and *R*_*T*_ are the region of tracking results and ground truth, and ⋂ and ⋃ are the intersection and union operations.

We have evaluated each tracker on 21 video sequences, which is recorded in our real experimental tests and the partial occlusion exists. For each video sequence, we run 15 times for each tracker and record the mean values of CLE in pixels and OP (%). Figure 8C quantitatively reports the comparative results of each tracking methods. Both the lowest value of mean CLE and the highest value of mean OP obviously indicate that ours is superior to others. Even the latest (Held et al., 2016), whose performance is well-acknowledged in computer vision benchmarks, underperforms when the target is partially occluded. The value of mean OP in our algorithm has sufficiently satisfied the requirement of robust tracking under partial occlusion.

To further validate the tracking performance under partial occlusion, our method is evaluated on the 29 sequences with the partial occlusion in the OTB-50 benchmark (Wu et al., 2013), in which attributes are fully annotated. We compare our method with the reported TOP 3 tracking algorithms in the benchmark using one-pass evaluation (OPE). The OPE uses the ground truth object location in the first frame and evaluates the tracker based on the average *precision score* or *success rate*.The former is the ratio of successful frames whose OR is larger than a given threshold to the total frames in a sequence, whereas the later is the percentage of frames whose CLE is less than a given threshold distance of the ground truth. Further, these success and precision curves are averaged over all the sequences to obtain the overall success and precision plots, respectively. The plots of OPE for the 4 trackers averaging over the OTB-50 sequences having occlusion are shown as Figure 8D. In (a), to achieve the same precision at 0.7 or more, our method demands less location error than others. In (b), the partial occlusion (overlap) is usually 30–70% when the gripper approaching to the bottom to grasp the object, and in this overlap interval, our method achieves higher success rate than others.

### 4.3. Validation of Proprioceptive Grasping

**Figure 9:**
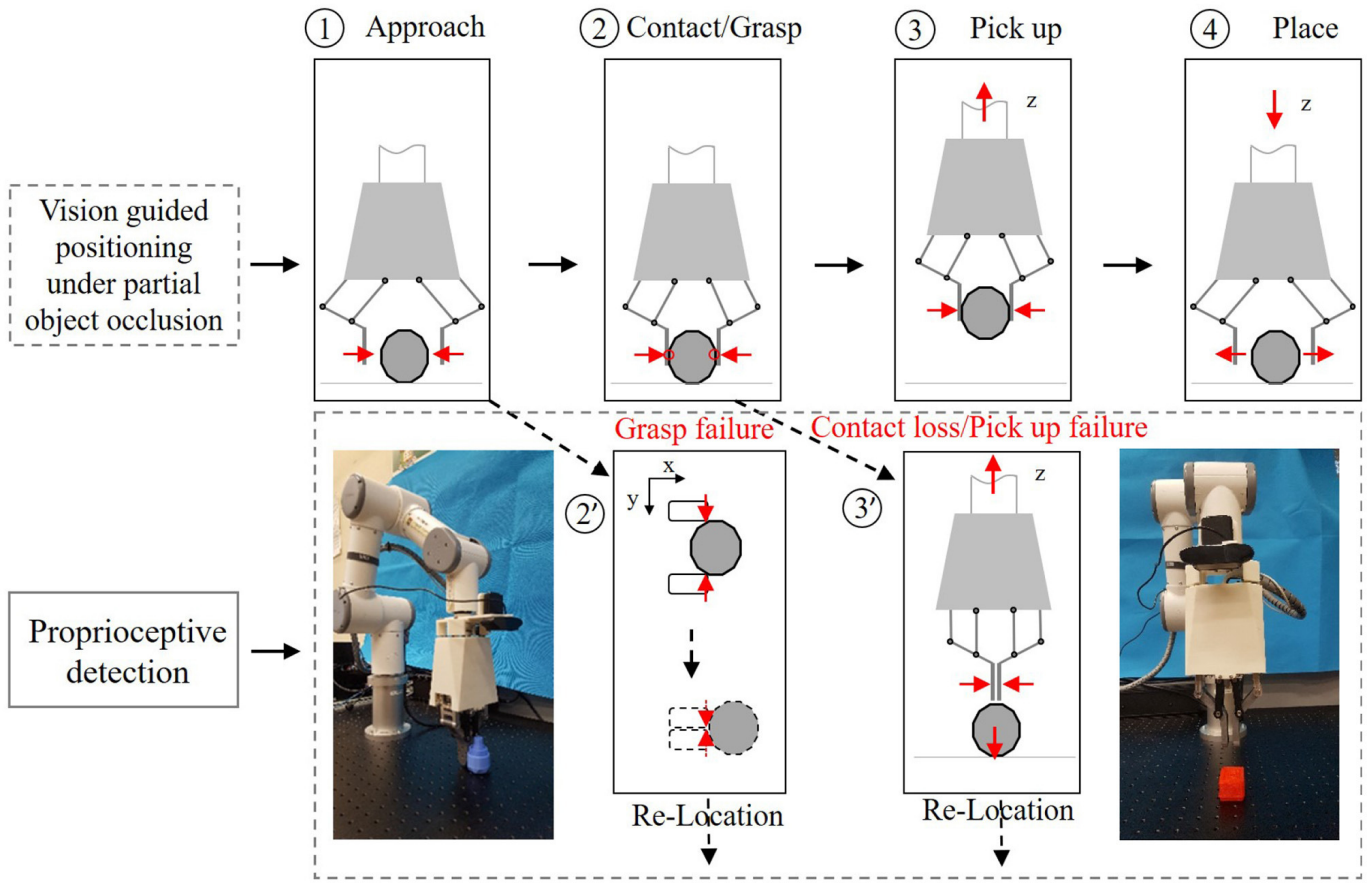
Pipeline of the object picking-and-placing task.

The pipeline of object picking and placing can be divided into four phases: (1) approach; (2) contact and grasp; (3) pick up; (4) place, as illustrated in Figure 9. First, the two-finger gripper approaches the target object with suitable pose configuration guided by vision. The two fingers then grasp the object with commanded grasping force. After that, the robot arm will pick up and place the object. It is very common in practice that the system may suffer task failure due to the disturbance in the environment, including visual position error or unstable interaction force. Proprioceptive grasp experiments were conducted using the two-finger gripper, including contact, grasping failure, and picking up failure detection.

#### 4.3.1. Contact Detection

**Figure 10:**
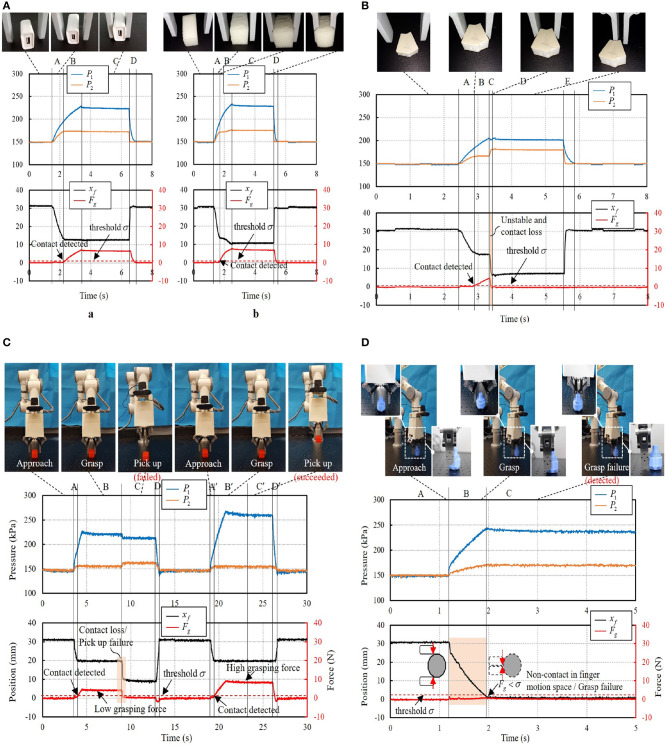
**(A)** Contact detection: *(a)* grasping of a rigid object; *(b)* grasping of a soft object. **(B)** Grasping failure due to unstable pinching. **(C)** Grasping failure due to visual positioning error. **(D)** Picking up failure due to small grasping force.

We validate contact detection on both rigid and soft objects. Figure 10A presents the recorded data of grasping a rigid object (Figure 10Aa) and a soft object (Figure 10Ab), reporting the pressures of the actuator *P*_1_(*t*),*P*_2_(*t*), finger position *x*_*f*_(*t*), and estimated grasping force *F*_*g*_(*t*). A constant threshold σ = 0.8*N* was set to trigger the contact detection signal. The system was capable of rapidly detecting the collision with the objects during the grasping. After the contact was detected, the finger motion would stop when grasping a rigid object while the fingers would keep its movement when grasping a soft object as can be seen in phase B in Figure 10A.

#### 4.3.2. Grasping Failure Detection

Figure 10B shows a case when grasping an irregular plane object. The object was successfully pinched at time *t* = 2.9 s when *F*_*g*_(2.9) > 0.8 N. But due to the unstable grasp, the object was popped up suddenly at time *t* = 3.3 s. Contact loss was then detected with Δ*F*_*g*_(*kT*) < −Δ(*k* = 3, Δ = 0.75*N*) and a grasping failure was recognized.

Figure 10C demonstrates another grasping of a bolt part with a cylinder surface. Due to inaccurate object positioning from the visual result, the object was slightly squeezed out from the two fingers against the cylinder surface. During the fingertip closing motion, non-contact event was triggered as the monitoring contact force *F*_*g*_(*t*) kept smaller than the threshold σ (σ = 0.8*N*). In this case, grasping failure was recognized with finger movement approaching the collision point (*x*_*f*_ = 0*mm*). As grasping “null” was detected, the robot arm stopped the picking up movement and instead to the relocation of the bolt part via the vision system.

#### 4.3.3. Picking Up Failure Detection

As shown in Figure 10D, the robot system was commanded to grasp a heavy cuboid part and pick it to the target place. From the vision result, no indication can be provided for how large the grasping force should be. Thus, the system commanded a small grasping force (*F*_*g*_ = 4*N*) and it succeeded in grasping the cuboid object. But it failed in the first trail of picking up the object due to insufficient grasping force. The monitoring contact force suffered a sudden decrease when the object slipped off from the two fingers. Contact loss was then detected and recognized as picking up failure with △*F*_*g*_(3*T*) < −0.75*N*). The robot arm stopped the picking up movement and relocation of the cuboid part proceeded. After relocating the cuboid part, the gripper was commanded with a larger grasping force (*F*_*g*_ = 8*N*) and succeeded in picking up the cuboid part in the second trial. In case it failed again, the aforementioned process may be continued until placing the cuboid part.

### 4.4. Validation of Efficiency Improvement After Failure Detection and Recovery

**Figure 11:**
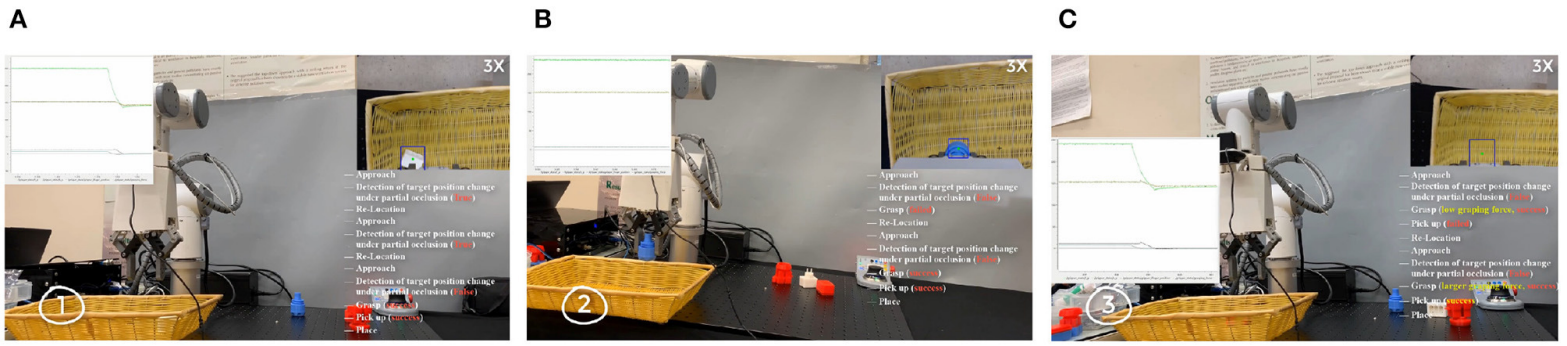
Experiments of the proposed three-phase failure detection in the real-time pick-and-place tasks. **(A)** Detection of target position changes. **(B)** Detection of grasping failure. **(C)** Detection of failures when picking up and moving to the destination.

We have designed the experimental tests to prove our failure detection and recovery system has improved the efficiency of robotic grasping (see Figure 11). With the same setup (as shown in Figure 2) and the same initial grasp planning (target detection and template retrieval) as our pipeline, the compared grasping system ignored the real-time failure detection and recovery, and no matter what failure occurs, the robot arm will complete the pick-and-place operation. If the former grasping is found failed by the worker or other assistive means, a new grasping needs to be planned for another complete pick-and-place operation until the grasping is finally successful.

When accidental failures occur in the grasping, we separately recorded the time–cost (the time from the 1^*st*^ grasp planning to the final successful object placing) of successfully grasping the object in Figure 9 with the pipeline without failure detection and recovery and ours in Figure 2. For each kind of failure, we separately did 20 tests in each pipeline. The average time–cost is 42.75 and 60.15 s separately for our pipeline and the pipeline without the failure detection and recovery. In our experiments, the average improvement of systematic efficiency is 40.7%.

## 5. Conclusion and Future Work

This paper presents an approach for effectively handling failures in the robotic pick and place task by combining multimodal cues under partial occlusion. We achieve more accurate pose estimation of a known object by considering the edge-based cost besides the image-based cost. Robust object tracking method is proposed to work even when the object is partially occluded and achieve mean OP up to 80%. Meanwhile, we take advantage of our proprioceptive soft gripper for the contact and contact loss detection by analyzing internal pressure signals of our gripper. With the combination of visual cues under partial occlusion and proprioceptive cues from our soft gripper, our system can effectively detect and recover from different failures in the entire procedure of robotic pick and place tasks.

To improve the accuracy of pose estimation, we introduced the edge-based cost besides the image-based cost. Meanwhile, a correlation-filter-based tracking approach is proposed to guaranteed the robustness of the grasping system even partial occlusion exists, especially when detecting and recovering from the failures. Yet, proprioception of our soft gripper is proved to be an effective complement to vision in physical interaction, facilitating the system to fast detect-response to dynamic disturbances, such as grasping failure and picking up failure. Experiments have validated the robustness and accuracy of our approaches.

In future work, more varieties of grasping targets will be explored, for example, the jelly-like objects that are non-rigid or dynamic objects. These are both potential targets in real applications. A more precise and closed collaboration of vision and proprioceptive cues will be required for this kind of grasping task. Meanwhile, the problem of target pose estimation is significant for deciding the gripper's pose in real-time robotic grasping. A more flexible and simplified method will be considered to determine the pose of the target by simply moving the camera to a specific position in 3D space and observing the static target in different camera views.

## Data Availability Statement

The original contributions presented in the study are included in the article/supplementary material, further inquiries can be directed to the corresponding author/s.

## Author Contributions

FZ developed the visual tracking techniques even with partial occlusion, completed corresponding validation, and wrote the first draft of the manuscript. LW extracted proprioceptive data and perform the statistical analysis. YW contributed to the object recognition and pose estimation in grasping. ZW and WW contributed to the conception and design of the experiments. All authors contributed to manuscript revision, and read and approved the submitted version.

## Conflict of Interest

The authors declare that the research was conducted in the absence of any commercial or financial relationships that could be construed as a potential conflict of interest.
